# Next‐generation studies of microbial biofilm communities

**DOI:** 10.1111/1751-7915.12390

**Published:** 2016-07-29

**Authors:** Scott A. Rice, Stefan Wuertz, Staffan Kjelleberg

**Affiliations:** ^1^ Singapore Centre for Environmental Life Sciences EngineeringNanyang Technological UniversitySingaporeSingapore; ^2^ School of Biological SciencesNanyang Technological UniversitySingaporeSingapore; ^3^ School of Civil and Environmental EngineeringNanyang Technological UniversitySingaporeSingapore

## Abstract

As we look into the future of microbial biofilm research, there is clearly an emerging focus on communities rather than populations. This represents an essential change in direction to more accurately understand how and why microorganisms assemble into communities, as well as the functional implications for such a life style. For example, current research studies shows that communities display emergent properties or functions that are not predicted from the individual single species populations, including elevated stress tolerance and resistance to antibiotics. Models for mixed species biofilms can be very simple, comprised only a handful of species or can be extremely species rich, with hundreds or thousands of species present. The future holds much promise for this area of research, where investigators will increasingly be able to resolve, at the molecular and biochemical levels, interspecies relationships and mechanisms of interaction. The outcome of these studies will greatly enhance our understanding of the ecological and evolutionary factors that drive community function in natural and engineered systems.

Biofilm research has advanced dramatically since its genesis as a discipline, when adhesion and colloid theory formed the foundation of the initial studies (Characklis and Marshall, 1990). The application of confocal microscopy to image living, hydrated, biofilms led to a revolution in biofilm research (Lawrence *et al*., 1991), This was followed by a range of molecular techniques including fluorescent *in situ* hybridization (FISH), localization of reporter gene expression, proteomics and transcriptomic‐based analyses to understand the molecular basis for biofilm formation and development. As a consequence, much has been learned about specific genes and proteins required for biofilm formation and development, biofilm stage‐specific gene expression, the division of labour during biofilm development, as well as the spatial and temporal localization of gene expression (Klausen *et al*., 2003; Labbate *et al*., 2004; Lenz *et al*., 2008). Due to technological limitations, these studies typically focused on single species systems.

We are currently witnessing a second revolution in microbial ecology and biofilm research, where advances in ‘omics’‐based technologies and computational sciences make it possible to study communities of microorganisms rather than simple populations. This is a critical step forward as it is well appreciated that almost no habitat contains a monoculture of bacteria, and biofilm development and function hence is a consequence of interactions between organisms. In recognition of this, several models of mixed species biofilms have been developed recently. Not surprisingly, many of these biofilms are characterized by relatively low complexity, typically consisting of two to four species. Nevertheless, these low diversity biofilms demonstrate that mixed communities are functionally distinct from single species biofilms, displaying emergent properties not predicted from biofilms formed by their individual member species. For example, Burmolle *et al*. (2006) showed that a mixed species biofilm achieves significantly more biomass than the monospecies biofilms (Ren *et al*., 2015), without the need to input more nutrients. Further, we and others have observed that such mixed communities have heightened tolerance to antimicrobials, chemical stress and predation (Burmolle *et al*., 2006; Lee *et al*., 2014; Kumar and Ting, 2015). Additional models allow for studies of autotroph–heterotroph interactions (Elenter *et al*., 2007; Cole *et al*., 2014), metabolic cooperation to degrade xenobiotics (Christensen *et al*., 2002) and cooperation and competition between different species (Foster and Bell, 2012; Fiegna *et al*., 2015), as examples of biofilm community interactions recently explored.

It will be vital for such models to expand in species and trophic‐level complexity and the types of organisms investigated. Measures of cooperation and competition along with quantified parameters of additional key traits, such as fitness and resilience, among others, require clear definitions of how those properties are being measured and the context in which they are being evaluated. It will also be essential to compare results under growth conditions that are relevant for the member species and their environment of origin. For example, mixed microbial communities associated with chronic infections of humans may be most reflective of their behaviour *in vivo*, when grown at 37°C in media that simulate the specific host environment. Similarly, we should endeavour to compare the results from different models to try to identify what constitute general principles of mixed species biofilms relative to the details that apply to a specific model system.

One key objective for such systems will be to establish measures of reproducibility such that more detailed studies of interactions can be undertaken with confidence. This is dependent on an increased awareness of the need for quantitative rather than qualitative analyses. The measure of reproducibility may need to be predicated on the model and key questions of interest, but can include species composition, structural organization and substrate utilization (or process performance). The question of reproducibility will also apply to more complex models of biofilm community interactions, which while more challenging, are arguably still tractable. For example, we have shown that an enrichment culture of a mixed microbial community can be used in repeated studies to foul water purification membranes, where the biofouling process and community composition are reproducible within certain limits (Barnes *et al*., 2015). Similarly, a highly species rich community undergoing granulation shows a strongly reproducible shift in community composition, driven by quorum sensing, highlighting that high complexity is not a barrier to reproducibility (Tan *et al*., 2015). This, then, allows for statistical analyses to be used with some degree of confidence. For example, it has been demonstrated that the community that develops in an anaerobic digester does so with sufficient reproducibility to show that there is strong selection for specific community members as opposed to a stochastic process of community assembly (Vanwonterghem *et al*., 2014). Specific lineages of *Candidatus Accumulibacter phosphatis* that were closely related to the Type II clade that dominated the microbial community capable of performing enhanced biological phosphorus removal in a full‐scale tropical wastewater treatment plant, as determined from long‐term monitoring and both 16S rRNA amplicon sequencing and whole‐cell FISH analyses (Law *et al*., 2016).

We contend that it is increasingly possible to ask similarly detailed quantitative questions for high complexity biofilms or communities. Hence, this allows us to understand the interactions of the member species based on community composition, physical organization and function. The ability to characterize such communities *in toto* would be the ultimate goal, such that all of the interacting partners are present and accounted for. By approaching the system as a whole, it may become possible to illuminate the function of the currently uncharacterized majority, often referred to as the microbial dark matter. Such studies would benefit from the development of a full cycle analysis, where genomic information allows for the identification of the community members and genetic potential coupled with their meta‐transcriptomic or proteomic complement.

The confirmation of activity and function of individual genes from the community requires meta‐omics‐based analyses. For example, using a ‘reverse metagenomics’ approach, we will be able to predict functions or regulation of genes and to verify those predictions through targeted cloning and gene evaluation. However, while meta‐omics studies are commonly reported, they remain limited by the amount of biomass required. As a consequence, most results represent averages across scales spanning vastly different microdomains, characterized by large differences in oxygen and nutrient concentrations. Thus, we inevitably need to develop procedures to capture subfractions of the biofilm or community and to sequence those without the need for PCR amplification. Stable isotope‐based methods can then be used to identify functional groups or organisms, and ultimately, imaging‐based methods can resolve spatial relations at scales relevant to microorganisms. The coupling of FISH with separation techniques, such as flow cytometry, would allow for key organisms to be separated and isolated for further study and potentially to reconstitute a minimally diverse community of key functional organisms.

With regard to community function, it is difficult to ascribe metabolites to specific organisms if those participate in common pathways. Therefore, it may be advantageous to consider metabolism as a community property and to develop metabolic network maps for such communities. This will elucidate energy fluxes and biochemical transformations that enable the community to achieve more efficient substrate utilization and to attain a higher overall biomass from the same amount of nutrient input relative to individual species. It is highly likely that this will also identify community‐level pathways that are distinct from classical central metabolism.

The extracellular polymeric substances (EPSs) that encase cells and glue them together in the biofilm are one of the defining hallmarks of a biofilm (Costerton *et al*., 1987). Despite this, EPS is typically treated as a passive component of the biofilm, where activity and function are solely based within the cells. EPS is very poorly defined in terms of both a detailed identification of the biopolymers involved and their function. Indeed, almost all papers say ‘… the EPS matrix is composed of polysaccharides, proteins and extracellular DNA …’ and the state‐of‐the art would be to indicate that the biofilm matrix of *Pseudomonas aeruginosa* comprises three polysaccharides, Psl, Pel and alginate. However, encoding the enzymatic machinery does not guarantee production nor does it indicate how much of each component, or whether polysaccharides in the EPS, change over time. We argue that the extracellular matrix consists of a range of polymeric constituents, which in addition to providing defined structural roles, also interact with and transport small molecules such as quorum sensing signals and redox shuttles for the orchestration of activities throughout the biofilm matrix. It is clear that the matrix, which makes up some 90% of the biofilm biovolume (Flemming and Wingender, 2010), is much more than just a sticky substance that holds cells together. Indeed, the matrix imbues the biofilm with a range of attributes, including antibiotic resistance, storage of extracellular enzymes, nutrient capture, gradient formation and protection from stress, that single cells or planktonic cells cannot achieve in its absence. In this way, the matrix is truly responsible for the emergent properties of the biofilm (Flemming *et al*., 2016).

Because the matrix plays such an important role for the biofilm, it is essential that tools and approaches are developed to define the specific matrix components, biochemically and structurally, as well as their physical properties. This is largely hampered by the lack of appropriate methods for the extraction of matrix materials, without contamination from cellular components. For example, the use of novel solvent systems, such as non‐ionic liquids, coupled with an understanding of solubility parameters has been used to extract high‐molecular‐weight matrix components (Seviour *et al*., 2015). New, quantitative methods or approaches to define the roles of individual matrix components will also help us to understand how the biofilm functions. For example, the rheological properties have been used to define the cohesive forces of the biofilm (Stoodley *et al*., 2002), and more recently, microrheology shows that biofilms are made up of different domains with unique viscoelastic properties and hence mechanical properties that change during biofilm development (Chew *et al*., 2014) and that the individual matrix components have a significant impact on mixed species community assembly (Periasamy *et al*., 2015). Further, it was recently shown that the presence of a filamentous bacteriophage can result in conversion of the matrix into liquid crystalline structure, which is linked to antibiotic resistance and the physical stability of the biofilm matrix (Secor *et al*., 2015). As the matrix components are identified, it may be possible to develop specific visualization approaches, such as the *Wisteria floribunda* lectin that specifically recognizes the Pel polysaccharide (Jennings *et al*., 2015), so that the localization and interactions between EPS components can be studied. This may also help explain why the biofilm is more than the sum of its individual parts. This highlights some of key points; the matrix may contain more than proteins, eDNA and polysaccharides; the interaction of different matrix materials will alter the physical properties of the EPS; and tools and methods from different fields, in particular that of biophysics, can be applied to biological systems to generate new information that helps to explain the properties of the biofilm matrix. These details will then enable a better understanding of how mixed species consortia assemble and organize themselves, where the matrix components may dictate who sticks best to whom, and where we may arrive at an understanding of matrix components that drive community effects, including both structural and functional traits.

With respect to mixed species community assembly and function, we must take advantage of the rich body of work and theory underpinning macro‐ecology. These approaches can be used to help provide explanations and models for microbial community assembly and function. For example, Vanwonterghem *et al*. (2014) predicted, based on neutral theory, that if microbial communities involved in anaerobic digestion were selected for based solely on function, then the final community composition should be highly variable, depending on changing dominance of different guilds. However, the results indicated that the communities within the anaerobic digestors were consistent at a species level, and thus the authors concluded that there must be other, strong selection forces at work within the reactors responsible for the community dynamic observed. Similarly, community composition and function have been described in terms of competition (Foster and Bell, 2012), cooperation (Ren *et al*., 2015), resource partitioning and the role of interspecific and intraspecific variation in population‐ and community‐level resilience (Lee *et al*., 2016). While microbiologists are increasingly embracing this approach, studies of molecular microbial ecology will greatly benefit not only from such an interdisciplinary uptake but ideally from the perspective of an experimental and theoretical platform where microbiologists and ecologists begin to unify macro‐ and micro‐ecology.

## Conflict of interest

None declared.
